# Sirt3 Exerts Its Tumor-Suppressive Role by Increasing p53 and Attenuating Response to Estrogen in MCF-7 Cells

**DOI:** 10.3390/antiox9040294

**Published:** 2020-04-01

**Authors:** Marija Pinterić, Iva I. Podgorski, Marijana Popović Hadžija, Vedrana Filić, Mladen Paradžik, Bastien Lucien Jean Proust, Ana Dekanić, Ivan Ciganek, Denis Pleše, Dora Marčinko, Tihomir Balog, Sandra Sobočanec

**Affiliations:** 1 Division of Molecular Medicine, Ruđer Bošković Institute, 10000 Zagreb, Croatia; mpinter@irb.hr (M.P.); iskrinj@irb.hr (I.I.P.); mhadzija@irb.hr (M.P.H.); Bastien.Lucien.Jean.Proust@irb.hr (B.L.J.P.); adekanic@irb.hr (A.D.); iciganek@stud.biol.pmf.hr (I.C.); dplese@pharma.hr (D.P.); dora.marcinko@krka.biz (D.M.); balog@irb.hr (T.B.); 2 Division of Molecular Biology, Ruđer Bošković Institute, 10000 Zagreb, Croatia; Vedrana.Filic.Mileta@irb.hr (V.F.); Mladen.Paradzik@irb.hr (M.P.); 3 Department Molecular Biotechnology and Health Sciences, Molecular Biotechnology Centre (MBC), University of Torino, 10124 Torino, Italy

**Keywords:** sirtuin 3, MCF-7, estrogen receptor, p53, breast cancer cells

## Abstract

Estrogen (E2) is a major risk factor for the initiation and progression of malignancy in estrogen receptor (ER) positive breast cancers, whereas sirtuin 3 (Sirt3), a major mitochondrial NAD^+^-dependent deacetylase, has the inhibitory effect on the tumorigenic properties of ER positive MCF-7 breast cancer cells. Since it is unclear if this effect is mediated through the estrogen receptor alpha (ERα) signaling pathway, in this study, we aimed to determine if the tumor-suppressive function of Sirt3 in MCF-7 cells interferes with their response to E2. Although we found that Sirt3 improves the antioxidative response and mitochondrial fitness of the MCF-7 cells, it also increases DNA damage along with p53, AIF, and ERα expression. Moreover, Sirt3 desensitizes cells to the proliferative effect of E2, affects p53 by disruption of the ERα–p53 interaction, and decreases proliferation, colony formation, and migration of the cells. Our observations indicate that these tumor-suppressive effects of Sirt3 could be reversed by E2 treatment only to a limited extent which is not sufficient to recover the tumorigenic properties of the MCF-7 cells. This study provides new and interesting insights with respect to the functional role of Sirt3 in the E2-dependent breast cancers.

## 1. Introduction

17β-estradiol (E2) is a steroid hormone essential for the maintenance of the female reproductive system with important physiological functions in the immune, cardiovascular, and neural systems [1,2]. However, E2 is a major risk factor for initiation and progression of malignancy in estrogen receptor (ER) positive breast cancers [3]. E2 mainly exerts its effect through the classical genomic pathway involving estrogen receptors alpha (ERα) and beta (ERβ) that function as transcription factors [4], with ERα being essential for proliferative signaling in both normal and breast cancer cells [5]. By binding to ERα, E2 promotes cellular proliferation through the upregulation of cell cycle regulating genes, and simultaneously triggers ERα proteasomal degradation required for the cellular response to environmental E2 levels [6]. 

Sirtuin 3 (Sirt3), NAD^+^ dependent deacetylase, is the only member of the sirtuin family that is linked to longevity in humans. In addition, important cellular and mitochondrial processes, including reactive oxygen species (ROS) generation, are integrated through Sirt3. It has been shown that Sirt3 has a bifunctional role in cancer, acting as both oncoprotein and tumor suppressor, depending on the tissue and cancer-type specific metabolic programs [7]. We have recently shown that Sirt3 has the inhibitory effect on tumorigenic properties of ERα positive breast cancer cells, particularly when combined with hyperoxic treatment [8]. However, it is not clear if this effect is mediated through the ERα signaling pathway. 

While ERα plays an important role in the progression of breast cancer, p53 functions as a major tumor suppressor through induction of target genes for cell cycle arrest and DNA repair [9]. Given that p53 functions primarily as a tumor suppressor, any aberration in the p53 gene or dysfunction of p53-mediated signaling pathway leads to cellular proliferation and potential tumorigenesis [10]. Breast cancer cells usually have functional p53, although its activity is altered by various mechanisms [11]. In ERα positive breast cancer cells, the abrogation of the p53 signaling pathway is a major event towards cancer progression, where p53 is functionally repressed by interaction with ERα [12]. However, the mechanism underlying the inactivation of p53 function is not fully understood. 

Although the elevated levels of ERα contribute to an increased risk of breast cancer [3], it has also been reported that ERα overexpression can be associated with reduced metabolic potential and invasiveness [13]. Earlier studies have shown that low Sirt3 expression is associated with reduced survival in all breast cancers and highlighted its potential role as a biomarker to assist in identifying high risk patients [14]. Furthermore, it was also shown that the full-length nuclear Sirt3 can indirectly activate and prevent degradation of p53 in MCF-7 cells through deacetylation of phosphatase PTEN [15]. Considering the tumor suppressive role of Sirt3, we hypothesized that E2/ERα signaling can be negatively affected by Sirt3. So far, it has not been shown that Sirt3 exerts its tumor-suppressive function in MCF-7 cells by interfering with their response to E2. 

Here, we report that overexpressed Sirt3 reduces the response of MCF-7 cells to E2, affects p53 by disruption of the ERα–p53 interaction, and inhibits the clonogenic growth of MCF-7 cells. Thus, Sirt3 can be considered to reduce tumor-initiating capacity of these cells by attenuating response of the ERα positive breast cancer cells to the E2. These results provide new and interesting insights concerning the functional role of Sirt3 in breast cancer and its therapeutic potential in hormone-positive breast tumors.

## 2. Materials and Methods

### 2.1. Cell Lines, Transfection, Treatments

The MCF-7 cell line was obtained from Public Health England (London, UK; ECACC 86012803), tested for mycoplasma contamination, and was grown in high glucose Dulbecco’s modified Eagle medium (DMEM, Sigma-Aldrich, St. Louis, MO, USA) with 10% fetal bovine serum (FBS, Capricorn Scientific, Germany), 1% nonessential amino acids (Sigma-Aldrich, St. Louis, MO, USA) and 1% antibiotic/antimycotic solution (Capricorn Scientific, Germany) at 37 °C with 5% CO_2_ in a humidified atmosphere. Due to the commercial source of the cells, there was no need to authenticate them prior to the study. The MCF-7 cells were transfected with the FLAG-tagged Sirt3 (MCF-7S3) or empty pcDNA3.1 plasmid (MCF-7C), as described previously in [8]. To examine the E2 effect on the characteristics of E2-dependent cell growth, we used white DMEM medium with steroid free (charcoal treated) serum (Sigma-Aldrich, St. Louis, MO, USA), since it is known that phenol red can activate ERα gene regulation [16,17]. Therefore, in our study, both phenol red and white DMEM (Capricorn Scientific, Germany) were used, depending on the parameters examined. For the experiments in white DMEM, cells were grown in this media for one week before the experiments. In our research, we treated cells with 10 nM E2 (17β-estradiol, Sigma-Aldrich, St. Louis, MO, USA) for 2 h and if we wanted to confirm that E2 effect was mediated by ERα, we treated cells with ERα inhibitor 100 nM ICI (ICI 182,780; Santa Cruz Biotechnology, Dallas, TX, USA) 2 h before adding E2.

### 2.2. RNA Isolation, Reverse Transcription, and qPCR Analysis

Total RNA was isolated from ~10^6^ cells using TRIzol reagent (Invitrogen, Carlsbad, CA, USA) according to the manufacturer’s instructions. Relative gene expression of *sirt-3* (Hs00953477_m1, TaqMan, Thermo Fisher Scientific, Waltham, MA, USA) and *esr-1* (Hs01046816_m1, TaqMan, Thermo Fisher Scientific, Waltham, MA, USA) were quantified by reverse transcription of total RNA and real-time quantitative PCR (qPCR) analysis. Data were analyzed using the 2^−ΔΔCt^ method and presented as the fold change in gene expression normalized to endogenous reference gene (*β-actin*; Hs01060665_g1, TaqMan, Thermo Fisher Scientific, Waltham, MA, USA) and relative to the control. All reactions were carried out in triplicate.

### 2.3. siRNA-Mediated Silencing of Sirt3 and ERα Expression

Silencing of Sirt3 and ERα expression was done using Lipofectamine2000 (Thermo Fisher Scientific, Waltham, MA, USA) according to the manufacturer’s guidelines. Sirt3 siRNA (siSirt3), ERα siRNA (siERα), and scrambled control siRNA (siSCR) were obtained from Ambion (Thermo Fisher Scientific, Waltham, MA, USA). Cells (10^5^) were seeded on a 24-well plate, and 24 h later transfected with 100 nM siRNA for 48 h, followed by protein collection and Western blot analysis for targeted proteins.

### 2.4. Fractionation, SDS-PAGE, and Western Blot Analysis

For fractionation analysis, 2 × 10^6^ cells were seeded on 10 cm Petri dish and 24 h later treated with ICI and E2, followed by fractionation by Cell Fractionation Kit Standard (ab109719, Abcam, UK) according to the manufacturer’s instructions. Proteins of obtained fractions were measured by Pierce™ BCA Protein Assay Kit (Thermo Fischer Scientific, Waltham, MA, USA) and prepared in SDS-PAGE sample buffer (100 mM Tris-HCl (pH 6,8), 2% SDS, 20% glycerol, 4% β-mercaptoethanol, 0.5% bromophenol blue dye) for Western blot analysis. Anti-GAPDH and anti-H3 antibodies were used as controls for purity of cytoplasmic and nuclear fractions, respectively [18,19]. Total cellular proteins for Western blot analysis were isolated in Ripa buffer with protease inhibitors (Roche, Basel, Switzerland). SDS-PAGE and Western blot analysis were carried out as described previously in [8]. Primary and secondary antibodies used in this study are listed in Supplementary Table S1. 

### 2.5. Immunofluorescence, Micronucleated Cells and Confocal Microscopy

Immunofluorescence analysis was performed as described previously in [20]. When MitoTracker Deep Red (Thermo Fisher Scientific, Waltham, MA, USA) was used, cells were labelled with 100 nM MitoTracker for 20 minutes before the end of the E2 treatment. Primary and secondary antibodies used in this study are listed in Supplementary Table S2. DAPI (4,6-diamidino-2-fenilindol, Sigma-Aldrich, St. Louis, MO, USA) was used for nuclear staining. For detection of micronucleated cells, 10^4^ cells/well were seeded on coverslips in a 24-well plate and were grown in white DMEM for 10 days, then fixed with 4% PFA, and stained with 5 µM Hoechst 33342 (Sigma-Aldrich, St. Louis, MO, USA) for 10 min. As a positive control, formation of micronucleated cells in MCF-7C clone was induced with 400 µM H_2_O_2_ for 4 h after which they were left to grow in fresh DMEM for the next 72 h, and then were further processed and analyzed as untreated cells. Confocal imaging was performed by sequential scanning using a Leica TCS SP8 X laser scanning microscope (Leica Microsystems, Germany), equipped with a HC PL APO CS2 63/1.40 oil immersion objective and a white light laser. The excitation wavelengths and emission detection ranges used were 350 nm and 412 to 460 nm for Hoechst 33342, 405 nm and 412 to 460 nm for DAPI, 488 nm and 495 to 550 nm for Alexa488, 594 nm and 601 to 644 nm for Alexa594, and 644 nm and 651 to 700 nm for MitoTracker Deep Red, respectively.

### 2.6. Cellular Proliferation, Metabolic Activity, Clonogenic Capacity

In order to determine the cell proliferation, EdU Click-iT^®^ assay (Thermo Fisher Scientific, Waltham, MA, USA) was used according to the manufacturer’s instructions [21]. Briefly, 4 × 10^5^ cells were seeded in six-well plates in both red and white DMEM, 24 h later they were treated with E2 and ICI, and left to grow for an additional 48 h. The samples were analyzed using FACS Calibur flow cytometer (BD Biosciences, Franklin Lakes, NJ, USA), while acquisition was made using the CellQuest software package (BD Biosciences, Franklin Lakes, NJ, USA). The analysis of the frequencies of proliferative (EdU positive) cells was performed using the FCS Express 3 software package (De Novo software, Pasadena, CA, USA). For the MTT(3-(4,5-dimethylthiazol-2-yl)-2,5-diphenyl tetrazolium bromide; tetrazolium dye) assay, 5 × 10^3^ cells were seeded in a 96-well plate and 24 h later treated with ICI and E2 and processed as described previously in [8]. For the clonogenic capacity (CFU) assay, 2 × 10^3^ cells were seeded in 5 cm Petri dishes, and 24 h later treated with ICI and E2. After that, the cells were incubated for 14 days until the visible colonies were observed and processed as previously described [8]. 

### 2.7. Migration Assay

For monitoring cell migration, 1 × 10^5^ cells were treated for 2 h with E2 in serum-reduced DMEM, seeded in migration Transwell Cell Culture Inserts (pore size 8 mm; Corning, Corning, NY, USA), and left to migrate for 22 h towards 10% FBS in DMEM as a chemoattractant. Cells migrated to the underside of the filter were fixed with 4% PFA, stained with 1% crystal violet solution, photographed, and quantified using NIH ImageJ (v1.52a, U.S. National Institutes of Health, Bethesda, MD, USA).

### 2.8. Measurements of Mitochondrial Membrane Potential, Cytosolic, and Mitochondrial ROS Production

Quantitative analysis of mitochondrial membrane potential (ΔΨm), and mitochondrial superoxide production (mtROS) was carried out using 100 nM MitoTracker Deep Red and 5 μM MitoSOX Red reagent (both from Thermo Fisher Scientific, Waltham, MA, USA), respectively. Cytosolic ROS production was measured with 20 μM dihydroethidium (DHE) (Invitrogen Molecular Probes, Carlsbad, CA, USA). Sytox Red (500 nM, Thermo Fisher Scientific, Waltham, MA, USA) and PI (1.5 μg/mL) were used for exclusion of dead cells dyed with MitoSOX Red or DHE, and MitoTracker Deep Red, respectively. The samples were analyzed using a FACS Calibur flow cytometer as described above.

### 2.9. Antioxidant Enzyme Activities

Superoxide dismutase (SOD) activity was assayed with a RANSOD kit (RANDOX Labs, UK) according to the manufacturer’s protocol. The SOD-2 activity was determined under identical conditions with the addition of 4 mM KCN in the assay buffer for 30 min to inhibit SOD1. The SOD1 activity was obtained by subtracting the SOD2 activity from the total SOD activity. Lyophilized cells and standard solutions were used for the SOD assay. The absorbance was measured at 505 nm on a microplate reader (Bio-Tek Instruments, Inc., Winooski, VT, USA).

### 2.10. Immunoprecipitation and Coimmunoprecipitation

Cells were seeded in white DMEM and 24 h later treated with E2 for 2 h, followed by cell lysis in Co-IP buffer (250 mM NaCl, 0,1% NP-40, 50 mM HEPES). Since Sirt3 is FLAG-tagged, ANTI-FLAG M2 affinity gel (Sigma-Aldrich, St. Louis, MO, USA) was used for Sirt3 coimmunoprecipitation. For ERα, anti-ERα antibody (F-10, Santa Cruz Biotechnology, Dallas, TX, USA) was used and Pierce Crosslink Immunoprecipitation Kit (Thermo Fisher Scientific, Waltham, MA, USA) according to the manufacturer’s guidelines, with the exception of the Co-IP buffer instead of IP Lysis/Wash Buffer from the kit. 

### 2.11. Statistical Analysis

Statistical analysis of data was performed using R v2.15.3 (CRAN, http://cran.r-project.org) and RStudio for Windows, v0.97 (http://www.rstudio.com/) and SPSS for Windows (17.0, IBM, Armonk, NY, USA). Before all analyses, the samples were tested for normality of distribution using the Shapiro–Wilk test. If the data followed a non-Gaussian distribution, the following nonparametric analyses were performed: Kruskal–Wallis non-parametric ANOVA for testing differences between groups that do not follow normal distribution, followed by Wilcoxon signed-rank test for testing differences between two related groups. In the case of normal distributions, the following parametric tests were performed: two-way ANOVA, followed by Bonferroni adjustments for the analysis of (simple) main effects. For comparisons of the two samples, the Student’s t-test or Mann–Whitney U test were used, depending on the distribution of data. Significance was set at *p* < 0.05.

## 3. Results

### 3.1. Sirt3 Participates in Regulation of ERα Expression and Localization and Alters Its Response to E2 Treatment in MCF-7 Cells

**Figure 1:**
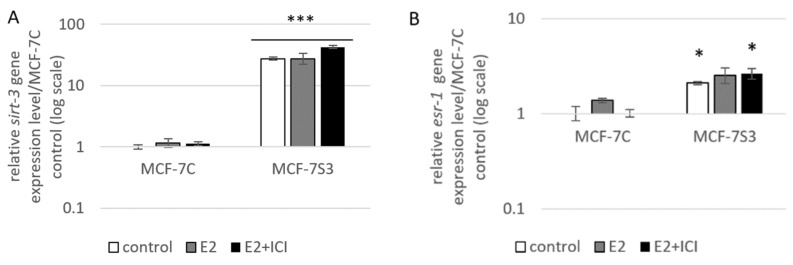

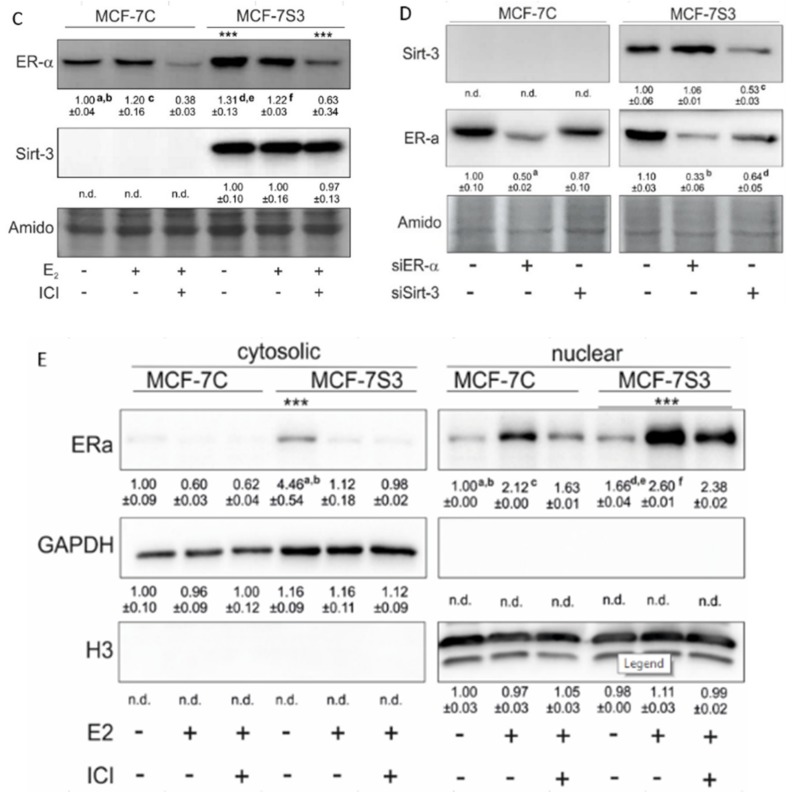
Sirt3 regulates ERα expression and localization. (**A**) Expression of *sirt-3* gene was significantly increased in MCF-7S3 clones as compared with MCF-7C (*** *p* < 0.001). The results are presented as fold change ± SE on a log-scale, normalized to the control MCF-7C. Experiments were repeated at least three times (hereafter referred to as *n* ≥ 3); (**B**) Expression of *esr-1* gene was significantly increased in the control and E2 + ICI-treated MCF-7S3 clones as compared with MCF-7C (* *p* < 0.05); (**C**) Immunoblots of ERα and Sirt3 protein expression level. Two-way ANOVA revealed significant interaction effect between Sirt3 and treatment on ERα F (2,12) = 9.321, *p* = 0.004, partial *η*^2^ = 0.608; higher in the control (*** *p* < 0.001) and E2 + ICI-treated (*** *p* < 0.001) MCF-7S3 vs. MCF-7C. In MCF-7C, higher in E2 vs. the control (^a^ *p* < 0.01) and E2 + ICI-treated (^c^ *p* < 0.001) and in the control vs. E2 + ICI-treated (^b^ *p* < 0.001). In MCF-7S3, higher in the control vs. E2 (^d^ *p* < 0.01) and E_2_ + ICI-treated (^e^ *p* < 0.001) and in E2 vs. E_2_ + ICI-treated (^f^ *p* < 0.001); (**D**) Immunoblots of ERα and Sirt3 protein expression level in the cells transfected with scrambled (siSCR) control or siER-α and siSirt3 RNA. Lower ERα in the siER-α-treated vs. siSCR MCF-7C cells (^a^ *p* < 0.05) and MCF-7S3 cells (^b^ *p* < 0.01). In MCF-7S3, lower Sirt3 (^c^ *p* < 0.05) and ERα (^d^ *p* < 0.01) in the siSirt3-treated cells; (**E**) Immunoblots of ERα protein expression in cellular fractions. For cytosolic fraction, two-way ANOVA revealed a significant interaction effect between Sirt3 and treatment F (2,12) = 54.61, *p* < 0.001, partial *η*^2^ = 0.948; higher cytosolic ERα in the control MCF-7S3 vs. MCF-7C (*** *p* < 0.001). In MCF-7S3, higher cytosolic ERα in the control vs. E2 (^a^ *p* < 0.001) and E2 + ICI-treated group (^b^ *p* < 0.001). For nuclear fraction, two-way ANOVA revealed significant interaction effect between Sirt3 and treatment F (2,12) = 1054.57, *p* < 0.001, partial *η*^2^ = 0.997; higher nuclear ERα in MCF-7S3 vs. MCF-7C (*** *p* < 0.001). In MCF-7C, lower nuclear ERα in the control vs. E2 (^a^ *p* < 0.001) and E2 + ICI-treated group (^b^ ***p*** < 0.001); higher nuclear ERα in E2 vs. E2 + ICI-treated group (^c^ *p* < 0.001). In MCF-7S3, lower nuclear ERα in the control vs. E2 (^d^ *p* < 0.001) and E2 + ICI-treated group (^e^ *p* < 0.001) and higher nuclear ERα in E2 vs. E2 + ICI-treated group (^f^ *p* < 0.001). For (**C**), (**D**) and (**E**) results are shown as a ratio of the mean ± SD normalized to the control MCF-7C (*n* ≥ 3). Amidoblack was used as a loading control.

**Figure 2:**
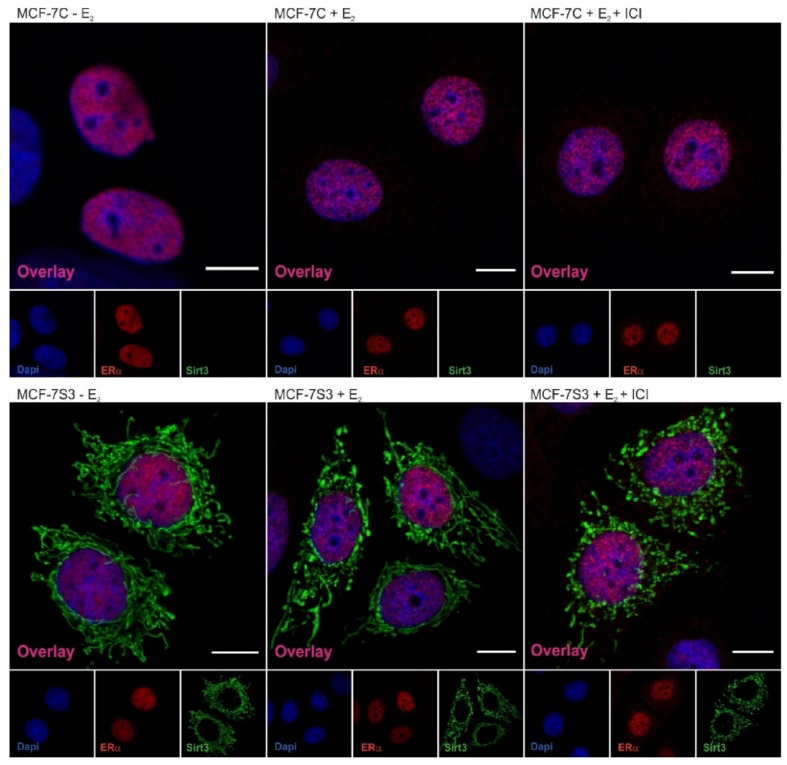
Sirt3 does not colocalize with ERα in MCF-7 cells. Confocal imaging of ERα localization in control, E2, and E2 + ICI-treated MCF-7C and MCF-7S3 cells. Bar represents 10 µm.

To examine if and how Sirt3 affects ERα expression, first, we stably overexpressed Sirt3 in MCF-7 cells (hereafter MCF-7S3) since MCF-7 cells express Sirt3 at almost undetectable levels (Figure 1A,C and Figure 2). Then, we analyzed mRNA and protein expression level of ERα in both MCF-7S3 and MCF-7 cells transfected with empty plasmid as a negative control (hereafter MCF-7C) (Figure 1B,C). Because MCF-7 cells proliferate in an E2-dependent manner [22], we next tested how E2 addition affected its cognate receptor ERα in the absence and presence of Sirt3. We found a positive effect of Sirt3 on both *esr-1* gene transcript and protein expression level, with +2.1-fold change and 30% increase in the absence of E2, respectively (Figure 1B,C). In the absence of Sirt3, E2 addition increased ERα protein expression (*p* < 0.01), whereas in Sirt3 clones it caused reduction of already upregulated ERα protein level (*p* < 0.01). Antiestrogen ICI, which was added two hours prior to E2 addition, had an inhibitory effect on ERα expression in both cell lines (Figure 1C, *p* < 0.001). We also examined the effects of Sirt3 or ERα silencing on ERα and Sirt3 expression (Figure 1D) and showed that Sirt3 silencing lowers the expression of ERα (*p* < 0.01). Furthermore, we investigated the effect of Sirt3 on cellular localization of ERα. Using the fractionation method, we found that E2 treatment promoted nuclear accumulation of ERα in both cell lines (*p* < 0.001), whereas a lower signal observed in cells treated with ICI prior to E2 addition indicated degradation of ERα. Interestingly, Sirt3 overexpression slightly delocalizes ERα in the cytosol (Figure 1E, *p* < 0.001). Confocal microscopy confirmed the primary localization of ERα in the nucleus (Figure 2). These results collectively indicate, while E2 affects both localization and the abundance of ERα expression, Sirt3 only affects the amount of ERα expressed in the cell.

### 3.2. Sirt3 Does Not Interact with ERα in MCF-7 Cells

Since the presence of ERα in mitochondria has been described by several studies [23,24], we next investigated the possibility that ERα resides inside mitochondria and interacts with Sirt3. Using both coimmunoprecipitation and confocal imaging, we found no interaction between ERα and Sirt3 (Supplementary Figure S1) or colocalization signal for ERα and Sirt3, respectively (Figure 2). These results collectively suggest that Sirt3 indirectly participates in the regulation of ERα expression.

### 3.3. Sirt3 Amplifies E2-Induced Metabolic Activity and Mitochondrial Fitness of MCF-7 Cells

**Figure 3:**
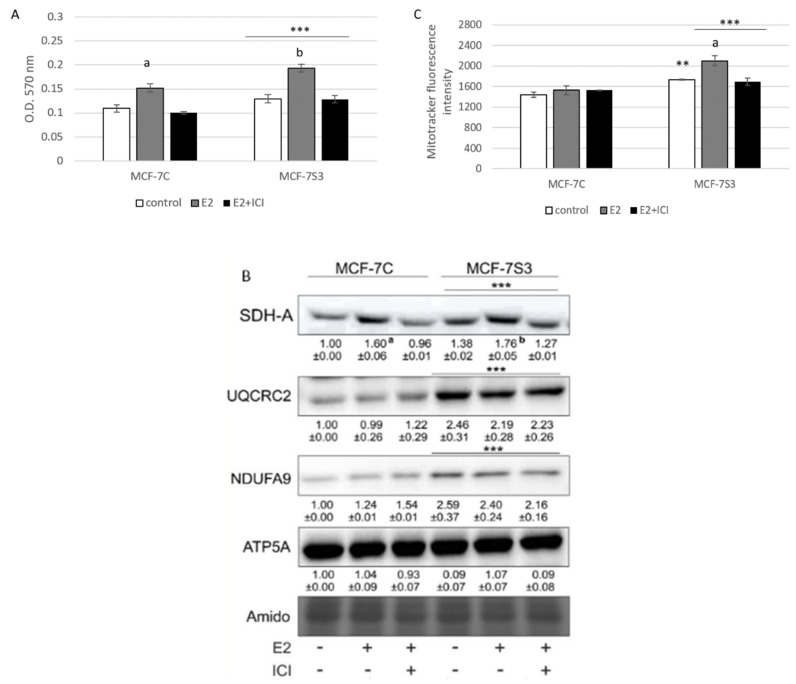
Sirt3 amplifies E2-induced metabolic activity and mitochondrial fitness of MCF-7 cells. (**A**) Metabolic activity measured with MTT assay. Two-way ANOVA revealed significant interaction effect between Sirt3 and treatment on metabolic activity F (2,34) = 7.051, *p* = 0.003, partial *η*^2^ = 0.293; *** *p* < 0.001 MCF-7S3 vs. MCF-7C. In MCF-7C, higher in E2 vs. other groups (^a^ *p* < 0.001). In MCF-7S3, higher in E2-treated vs. other groups (^b^ *p* < 0.001). Results are shown as mean ± SD (*n* ≥ 3); (**B**) Immunoblots of mitochondrial respiration complexes. Two-way ANOVA revealed significant interaction effect between Sirt3 and treatment on SDH-A expression F (2,6) = 10,846, *p* = 0.010, partial *η*^2^ = 0.783; *** *p* < 0.001 MCF-7C vs. MCF-7S3 cells. In MCF-7C, higher SDH-A expression in E2-treated vs. other groups (^a^ *p* < 0.001). In MCF-7S3, higher SDH-A expression in E2-treated vs. other groups (^b^ *p* < 0.001). Higher UQCRC2 and NDUFA9 protein expression in MCF-7S3 cells as compared with MCF-7C (*** *p* < 0.001). Results are shown as mean ± SD normalized to the control MCF-7C (*n* ≥ 3). Amidoblack was used as a loading control and representative immunoblots are shown; (**C**) Mitochondrial membrane potential (ΔΨm) measured with MitoTracker Deep Red dye using flow cytometry. Two-way ANOVA revealed significant interaction effect between Sirt3 and treatment F (2,12) = 11,019, *p* = 0,010, partial *η*^2^ = 0.786; higher ΔΨm in the control (** *p* = 0.003), E2, and E2 + ICI-treated (*** *p* < 0.001) MCF-7S3 vs. MCF-7C. In MCF-7S3, higher ΔΨm in E2 vs. the control and E2 + ICI-treated (^a^ *p* < 0.001). Results are shown as mean ± S.D (*n* ≥ 3).

To explore if Sirt3 plays a role in E2-induced metabolic activity of MCF-7 cells [25], we measured several parameters of mitochondrial function. First, we tested the effect of E2 and Sirt3 on metabolic activity of MCF-7 cells using MTT assay. The MTT salt is reduced to formazan in the metabolically active cells predominantly by mitochondrial complex-II subunit succinate dehydrogenase A (SDH-A) and is considered to be a marker of metabolic potential of the cell [26]. The Sirt3-overexpressing cells had significantly higher basal metabolic activity (*p* < 0.001) and Sirt3 further enhanced the inducing effect of E2 (Figure 3A), while ICI effectively abolished E2-induced metabolic activity to control levels in both cell lines. This result was confirmed with the observed protein expression levels of SDH-A (Figure 3B), indicating the combined effect of E2 and Sirt3 and involvement of ERα in the regulation of metabolic activity in MCF-7 cells. Furthermore, the expression levels of respiratory complex I (NDUFA9) and III (UQCRC2) were also elevated in Sirt3 overexpressors (*p* < 0.001), although with no significant effect of E2 (Figure 3B). Another marker of mitochondrial functionality is the mitochondrial membrane potential (ΔΨm) which plays a key role in mitochondrial homeostasis [27]. While in the absence of Sirt3, ΔΨm remained unaffected by E2 or ICI addition, the MCF-7S3 cells exhibited increased basal ΔΨm (*p* = 0.003), which was further enhanced by E2 addition and abolished by ICI (Figure 3C, *p* < 0.001). These results collectively suggest that (a) Sirt3 acts synergistically with E2 to induce metabolic activity, (b) E2-induced rise in mitochondrial potential is Sirt3 dependent, and (c) ERα is involved in Sirt3-mediated mitochondrial fitness of MCF-7 cells.

### 3.4. Sirt3 Enhances Antioxidative Enzyme Activities and Cytosolic ROS, but Opposes E2-Induced Cytosolic and mtROS Production

**Figure 4:**
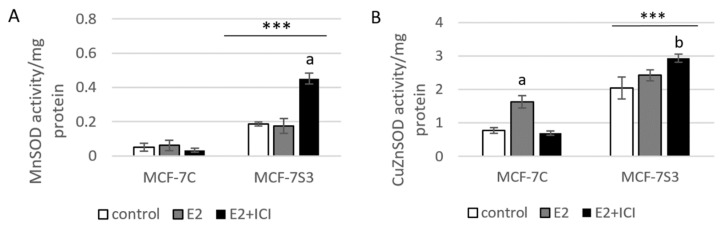

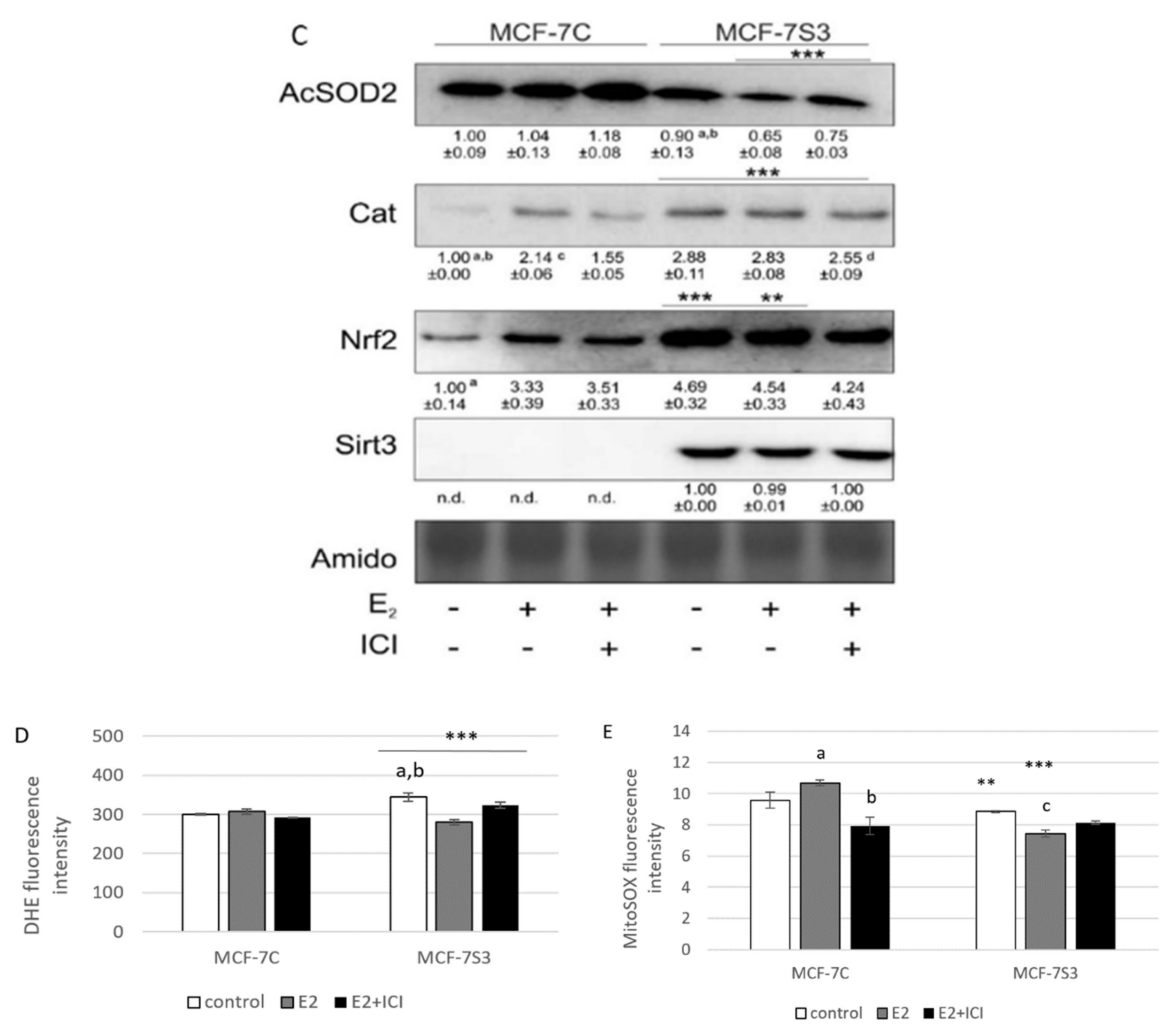
Sirt3 enhances antioxidative enzyme activities and cytosolic ROS but opposes E2-induced cytosolic and mtROS production. (**A**) For MnSOD activity, two-way ANOVA revealed a significant interaction effect between Sirt3 and treatment F(2,12) = 53,853, *p* < 0.001, partial *η*^2^ = 0.900; *** *p* < 0.001 MCF-7S3 vs. MCF-7C. In MCF-7S3, higher activity in the E2 + ICI-treated vs. the other groups (^a^ *p* < 0.001); (**B**) For CuZnSOD activity, two-way ANOVA revealed a significant interaction effect between Sirt3 and treatment F (2,12) = 75,435, *p* < 0.001, partial *η*^2^ = 0.952; *** *p* < 0.001 MCF-7S3 vs. MCF-7C. In MCF-7C, higher activity in the E2-treated vs. the other groups (^a^ p < 0.001). In MCF-7S3, higher activity in E2 + ICI vs. other groups (^b^ *p* < 0.001). Results are shown as mean ± SD (*n* ≥ 3); (**C**) For immunoblots of proteins of antioxidative response, two-way ANOVA revealed significant interaction effect between Sirt3 and treatment on AcSOD2 F (2,12) = 11,691; *p* < 0.01, partial *η*^2^ = 0.916; E2 and E2 + ICI-treated MCF-7S3 vs. MCF-7C (*** *p* < 0.001). In MCF-7S3, higher AcSOD2 in the control vs. the E2 (^a^ *p* < 0.01) and E2 + ICI-treated (^b^ *p* < 0.05). Two-way ANOVA revealed significant interaction effect between Sirt3 and treatment on Cat F (2,12) = 69,293, *p* < 0.001, partial *η*^2^ = 0.959; *** *p* < 0.001 MCF-7S3 vs. MCF-7C. In MCF-7C, lower Cat in the control vs. E2 (^a^ *p* < 0.001) and E2 + ICI-treated (^b^ *p* < 0.01); higher Cat in the E2 vs. E2 + ICI-treated (^c^ *p* < 0.01). In MCF-7S3, lower Cat in the E2 + ICI-treated vs. other groups (^d^ *p* < 0.01). Two-way ANOVA revealed significant interaction effect between Sirt3 and treatment on Nrf2 F (2,12) = 14,011, *p* = 0.005, partial *η*^2^ = 0.955; the control (*** *p* < 0.001) and E2-treated (** *p* < 0.01) MCF-7S3 vs. MCF-7C. In MCF-7C, lower Nrf2 in the control vs. the other groups (^a^ *p* < 0.01). Results are shown as mean ± SD normalized to the control MCF-7C (*n* ≥ 3). Amidoblack was used as a loading control; (**D**) For cytosolic ROS levels measured with DHE, two-way ANOVA revealed a significant interaction between Sirt3 and treatment F (2,12) = 805,710, *p* < 0.001, partial *η*^2^ = 0.996; *** *p* < 0.001 MCF-7C vs. MCF-7S3 cells. In MCF-7S3, lower cytosolic ROS in E2 (^a^ *p* < 0.001) and E2 + ICI-treated (^b^ *p* < 0.01) vs. the control cells; (**E**) For MtROS levels measured with MitoSOX Red, two-way ANOVA revealed a significant interaction effect between Sirt3 and treatment F (2,12) = 94.860, *p* < 0.001, partial *η*^2^ = 0.941; the control (** *p* = 0.002) and E2-treated (*** *p* < 0.001) MCF-7S3 vs. MCF-7C. In MCF-7C, higher mtROS in E2-treated vs. the control (^a^ *p* < 0.001); lower mtROS in E2 + ICI-treated vs. other groups (^b^ *p* < 0.001). In MCF-7S3, lower mtROS in E2-treated vs. other groups (^c^ *p* < 0.01). Results show the relative fluorescence intensity as the average geometric mean ± SD (*n* ≥ 3).

Since it is known that Sirt3 mediates mitochondrial oxidative pathways and regulates production of ROS (reviewed by [28]), we aimed to analyze the antioxidant enzyme system in MCF-7S3 cells. We found that MCF-7S3 cells exhibited significantly increased activities of two major antioxidative enzymes, mitochondrial manganese-dependent superoxide dismutase MnSOD (SOD2, Figure 4A, *p* < 0.001) and cytosolic copper zinc-dependent superoxide dismutase CuZnSOD (SOD1, Figure 4B, *p* < 0.001), which was also confirmed by decreased expression level of inactive, acetylated form of MnSOD, AcSOD2 (Figure 4C, *p* < 0.001). Moreover, protein levels of the catalase (Cat) and transcription nuclear factor erythroid 2-related factor 2 (Nrf2), a major activator of antioxidant response, were also upregulated in Sirt3-overexpressing cells (Figure 4C, *p* < 0.001). Next, we examined the role of E2 in the activation of antioxidant enzyme system. While having a positive effect on the activation of CuZnSOD (*p* < 0.001), Cat (*p* < 0.001), and Nrf2 (*p* < 0.01) in the control cells, in Sirt3 overexpressors, E2 failed to further increase their already elevated levels (Figure 4B,C). Collectively, these results indicate that Sirt3-overexpressing cells exhibit a higher antioxidative response regardless of the E2 treatment. Due to the elevated antioxidant enzyme system in the MCF-7S3 cells, we aimed to investigate the effect of Sirt3 and E2 on cellular ROS levels and, on the one hand, found that E2 promoted mitochondrial ROS (mtROS) production in MCF-7C cells (*p* < 0.001), and this effect was inhibited by ICI (Figure 4E, *p* < 0.001). On the other hand, both cytosolic and mtROS levels were significantly reduced in E2-treated MCF-7S3 cells as compared with their controls (Figure 4D,E and *p* < 0.001). However, without E2 treatment, MCF-7S3 cells exhibited a significant rise in cytosolic ROS (*p* < 0.001) with a parallel decline in mtROS levels (*p* = 0.002). These results indicate that Sirt3 increases cytosolic ROS but abolishes E2-induced increase of both cytosolic and mtROS levels.

### 3.5. Sirt3 Abolishes the Proliferative Effect of E2 on Colony Forming Capacity by Diminishing the E2-Induced DNA Synthesis of MCF-7 Cells

**Figure 5:**
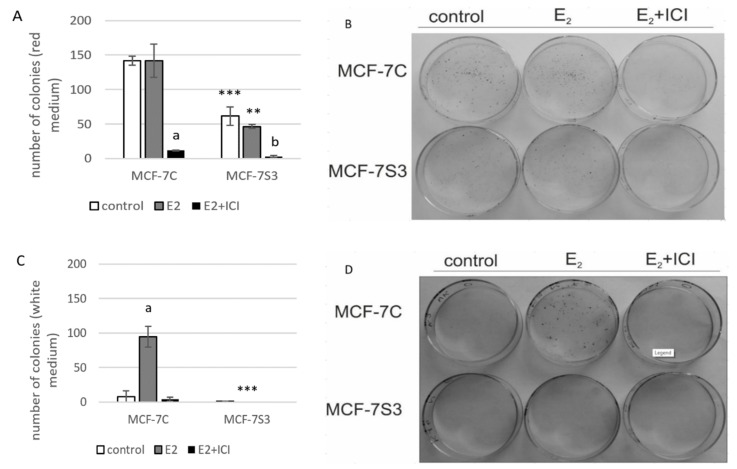

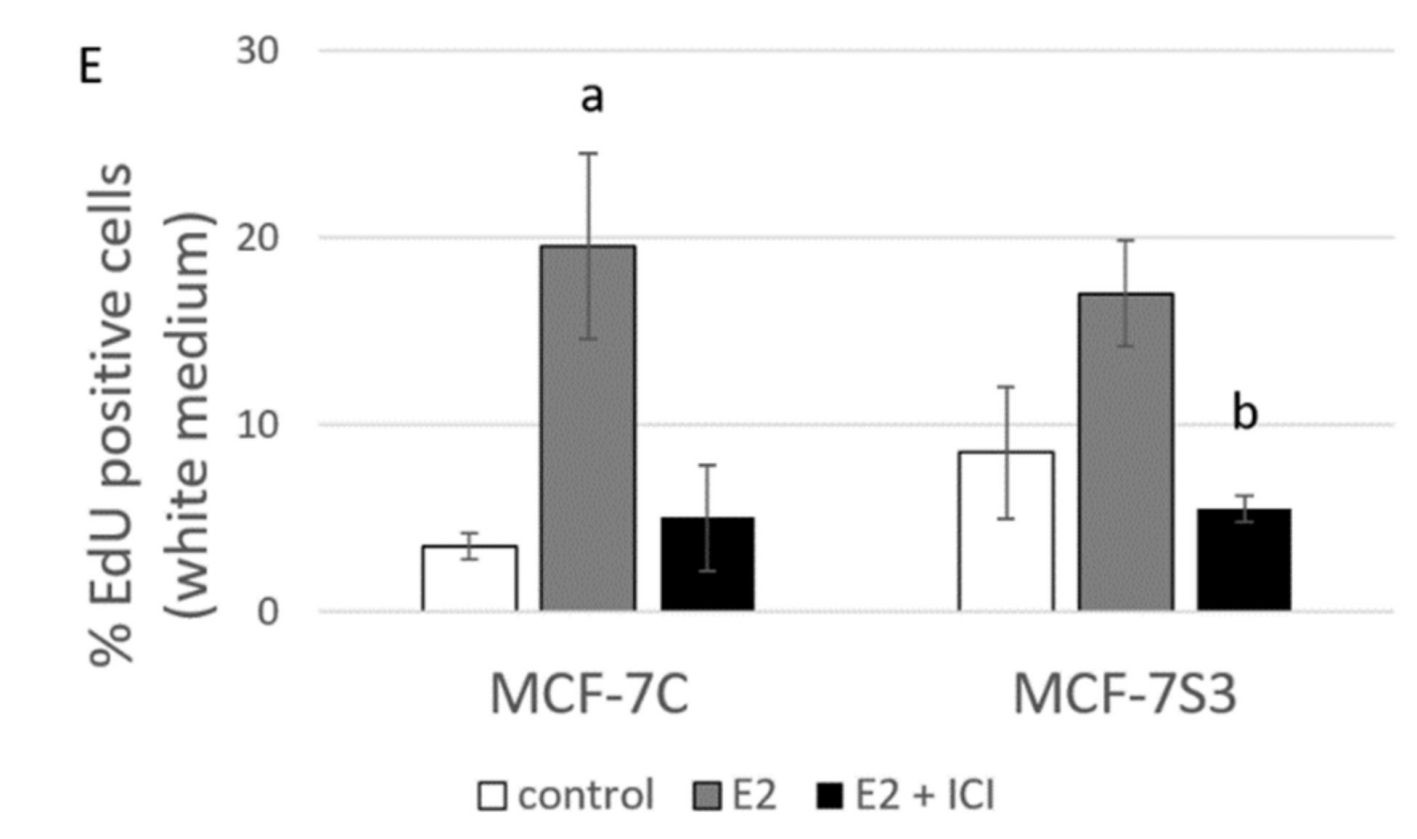
Sirt3 abolishes the proliferative effect of E2 on colony forming capacity by diminishing the E2-induced DNA synthesis of MCF-7 cells. (**A**) Histogram showing the number of colonies in red DMEM (CFU assay). Two-way ANOVA revealed significant interaction effect between Sirt3 and treatment on cellular clonogenic capacity F (2,15) = 26,780; *p* < 0.001; partial *η*^2^ = 0.856; the control (*** *p* < 0.001) and E2-treated (** *p* < 0.01) MCF-7S3 vs. MCF-7C. In MCF-7C, lower E2 + ICI-treated vs. other groups (^a^ *p* < 0.001). In MCF-7S3, the lower E2 + ICI-treated vs. the other groups (^b^ *p* < 0.001). Results are shown as mean ± SD (*n* ≥ 3); (**B**) Representative plates of colonies grown in red DMEM stained with crystal violet; (**C**) Histogram showing the number of colonies in white DMEM (CFU assay). Two-way ANOVA revealed a significant interaction effect between Sirt3 and treatment on cellular clonogenic capacity F (2, 6) = 52.397, *p* < 0.001, partial *η*^2^ = 0.946, E2-treated (*** *p* < 0.001) MCF-7S3 vs. MCF-7C. In MCF-7C, higher in the E2-treated vs. the other groups (^a^ *p* < 0.001). Results are shown as mean ± SD (*n* ≥ 3); (**D**) Representative plates of colonies grown in white DMEM stained with crystal violet; (**E**) DNA synthesis as a sign of proliferation in white DMEM measured with Click-iT^®^ assay using flow cytometry. In MCF-7C, higher in the E2-treated vs. other groups (^a^ *p* < 0.05). In MCF-7S3, lower in the E2 + ICI-treated vs. the E2-treated group (^b^ *p* < 0.05). Results are shown as mean ± SD (*n* ≥ 3).

Our previous results showed that Sirt3 has an inhibitory role on several tumorigenic parameters of MCF-7 cells [8], therefore, we wanted to analyze its effect on the colony forming capacity in the presence or absence of E2. Since the MCF-7S3 cells were not able to produce colonies in white DMEM, we also performed this experiment in red DMEM with the addition of E2 and ICI. The observed difference in clonogenic capacity of the cells in red and white DMEM is not surprising, since it is well known that ERα-positive breast cancer cells grow slower in phenol red-depleted media [17]. We observed a significant decrease in the colony forming ability of the MCF-7S3 cells in red DMEM as compared with the MCF-7C cells (Figure 5A,B and *p* < 0.001). As expected for the red DMEM, in which the phenol red mimics the action of E2 and renders cells unresponsive to the proliferative effect of physiological concentrations of E2 [29], E2 addition failed to show any difference in the number of colonies as compared with the untreated cells. In white DMEM, the colony forming capacity of the MCF-7C cells was reduced to 5% of their counterparts in red DMEM (Figure 5C,D). While E2 addition potentiated the growing capacity of the MCF-7C cells to nearly 70% of the cells in red DMEM, the MCF-7S3 cells completely failed to form colonies, even with the addition of E2. The number of colonies treated with ICI declined in both media as compared with their corresponding controls (*p* < 0.001). Collectively, these results indicate that Sirt3 attenuates the colony forming capacity of MCF-7 cells and abolishes the proliferative effect of E2 on MCF-7 cells. 

Due to the observed reduced capacity of Sirt3-overexpressing cells to divide and form colonies, we investigated if Sirt3 affects cellular growth by inhibition of DNA synthesis using EdU Click-IT^®^ assay (Thermo Fisher Scientific, USA). In white DMEM, E2 significantly promoted DNA synthesis in MCF-7C cells only (Figure 5E and *p* < 0.05), whereas the ICI treatment effectively inhibited DNA synthesis in both cell lines (*p* < 0.05). These data collectively indicate that Sirt3 abolishes cellular proliferation by inhibiting E2-induced DNA synthesis.

### 3.6. Sirt3 Induces Tumor-Suppressive Markers in MCF-7 Cells

**Figure 6:**
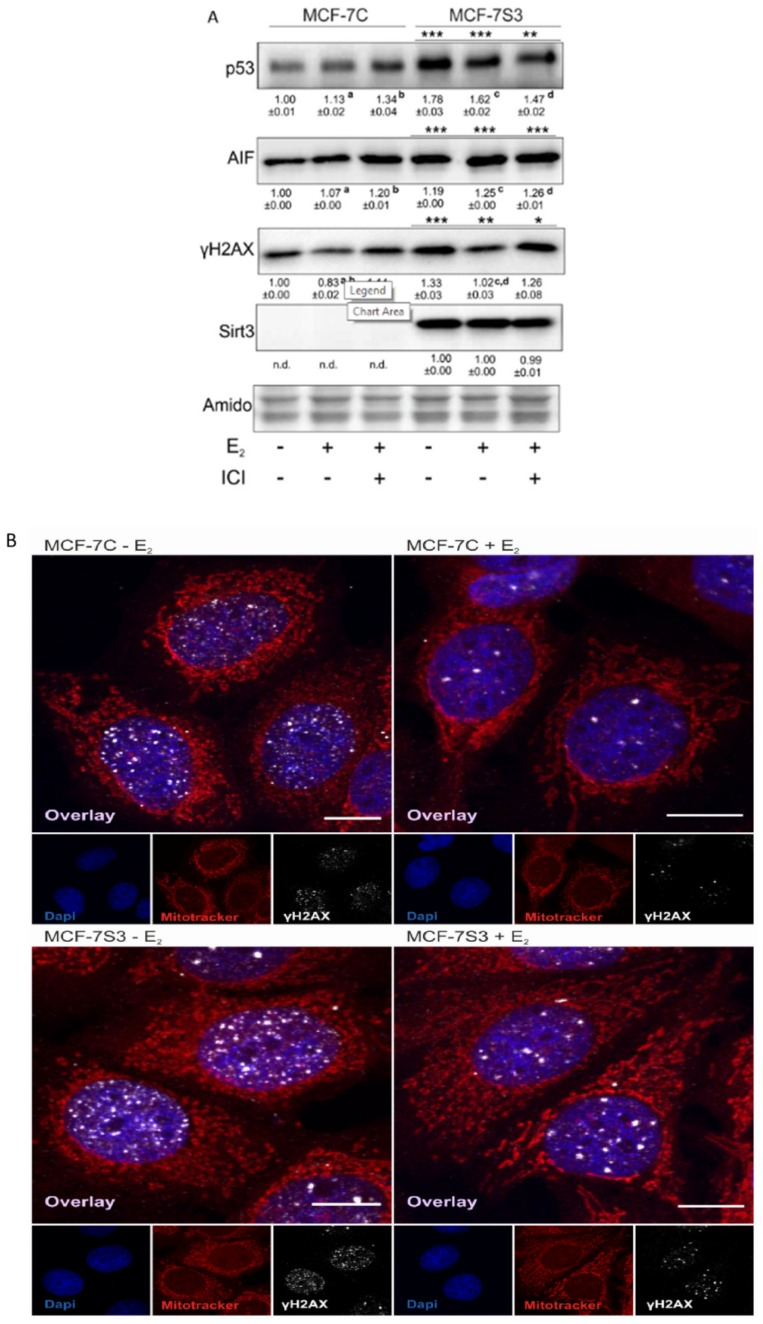

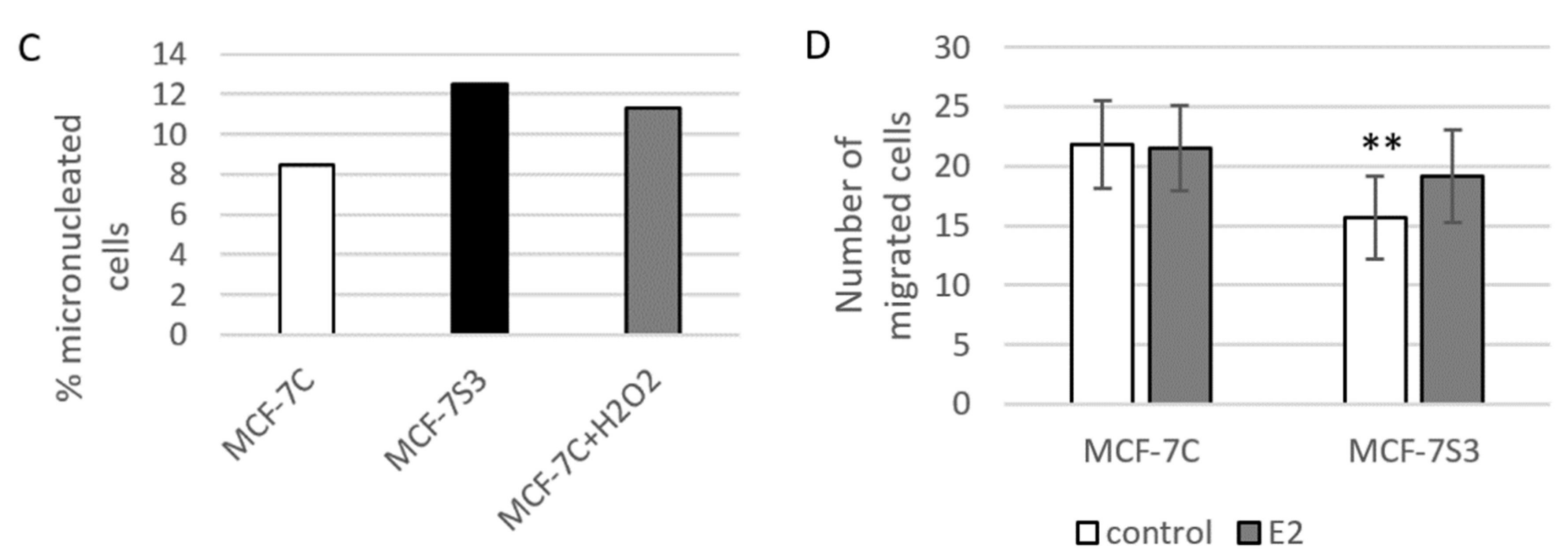
Sirt3 induces tumor-suppressive markers in MCF-7 cells. (**A**) Western blot analysis of proteins associated with tumor-suppressive function. Two-way ANOVA revealed a significant interaction effect between Sirt3 and treatment on the p53 level F (2,12) = 118.1, *p* < 0.001, partial *η*^2^ = 0.975; higher in the MCF-7S3 control and E2-treated (*** *p* < 0.001) and E2 + ICI-treated (** *p* < 0.01) vs. MCF-7C. In MCF-7C, higher in the E2 (^a^ *p* < 0.05) and E2 + ICI-treated (^b^ *p* < 0.001) vs. the control. In MCF-7S3, lower in the E2 and E2 + ICI-treated vs. the control (^c,d^ *p* < 0.01). For AIF, two-way ANOVA revealed a significant interaction effect between Sirt3 and treatment F (2,12) = 187.56, *p* < 0.001, partial *η*^2^ = 0.984; *** *p* < 0.001 MCF-7S3 vs. MCF-7C. In MCF-7C, higher in the E2 and E2 + ICI-treated vs. the control (^a,b^ *p* < 0.001). In MCF-7S3, higher in the E2 and E2 + ICI-treated vs. the control (^c,d^ *p* < 0.001). For phospho-γH2AX, two-way ANOVA revealed significant interaction effect between Sirt3 and treatment F (2,12) = 5.39, *p* < 0.05, partial *η*^2^ = 0.642; the MCF-7S3 control (*** *p* < 0.001), E2 (** *p* < 0.01), and E2 + ICI-treated (* *p* < 0.05) vs. MCF-7C. In MCF-7C, lower in E2 vs. the control (^a^ *p* < 0.05) and E2 + ICI-treated (^b^ *p* < 0.01). In MCF-7S3, lower in E2 vs. the control (^c^ *p* < 0.001) and E2 + ICI-treated (^d^ *p* < 0.01). Results are shown as mean ± SD (*n* ≥ 3). Amidoblack was used as a loading control; (**B**) Confocal imaging of phospho-γH2AX signal abundance as a marker of DNA damage in MCF-7C and MCF-7S3 cells with or without E2 treatment. Bar represents 10 µm; (**C**) Graphical chart of frequency of micronucleated cells in MCF-7C and MCF-7S3 cells, as well as in MCF-7C cells treated with H_2_O_2_ as a positive control. The experiments were repeated at least three times and representative data are shown; (**D**) Graphical chart of number of migrated MCF-7C and MCF-7S3 cells with or without E2 treatment. Two-way ANOVA revealed a significant effect of Sirt3 on migration rate F (1,23) = 1.812, *p* = 0.007, partial *η*^2^ = 0.279; lower in MCF-7S3 vs. MCF-7C cells (** *p* = 0.007). Results are shown as mean ± SD (*n* ≥ 3).

Since in our earlier study we observed higher levels of p53 in Sirt3-overexpressing cells [8], in this study, we investigated the level of p53 expression upon E2 treatment along with the expression of other p53-related proteins, such as apoptosis-inducing factor (AIF) and marker of DNA double strand breaks (phospho-γH2AX). Consistent with our previous results, the p53 level was elevated in MCF-7S3 cells (*p* < 0.001). However, E2 and ICI had the opposite effect on the p53 expression level in Sirt3-overexpressed and control cells; while both treatments decreased p53 expression in MCF-7S3 cells (*p* < 0.01), they elevated the expression in the control MCF-7C cells (Figure 6A, *p* < 0.05 for E2 and *p* < 0.001 for E2 + ICI). AIF was significantly increased by both E2 and Sirt3 (*p* < 0.001), whereas DNA damage showed to be higher in the presence of Sirt3 (*p* < 0.001), however, was lowered by E2 addition (*p* < 0.001). This was confirmed by confocal microscopy which clearly demonstrated more DNA damage in the Sirt3-overexpressed cells, and less in the E2-treated groups of both cell lines (Figure 6B). In addition, we also noticed a higher number of micronucleated cells in the Sirt3-overexpressed line (Figure 6C), which is considered to be an indicator of genomic instability and usually predisposes cells to apoptosis [30]. The MCF-7S3 line displayed a higher frequency of micronucleated cells than the controls, with values similar to the H_2_O_2_-treated cells, which were used as a positive control for the induction of apoptosis [31]. Since earlier studies showed that upon both DNA damage and p53 activation, cell migration is decreased [32,33,34], we also checked the migration of the cells. The migration rate was reduced in the MCF-7S3 cells as compared with the controls (*p* = 0.007). Moreover, while migration was not affected by E2 in the control cells, E2 administration only slightly increased migration in the Sirt3 overexpressors (Figure 6D and Supplementary Figure S2). These data collectively demonstrate the tumor-suppressive role of Sirt3 in MCF-7 cells, which is partially restored by E2 treatment.

### 3.7. Sirt3 Induces Disruption of ERα–p53 Interaction in MCF-7 Cells

**Figure 7:**
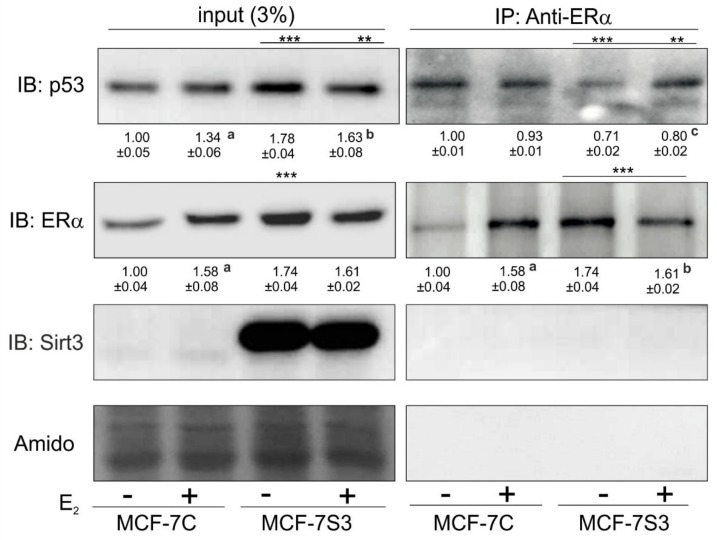
Sirt3 induces disruption of the ERα–p53 interaction in MCF-7 cells. Western blot analysis of coimmunoprecipitation experiment of ERα and its interacting partners using anti-ERα and anti-p53 antibody. For input p53, MCF-7C + E2 vs. MCF-7C (^a^ *p* < 0.001), MCF-7S3 + E2 vs. MCF-7S3 (^b^ *p* < 0.01), the MCF-7S3 control vs. MCF-7C (*** *p* < 0.001), MCF-7S3 + E2 vs. MCF-7C (** *p* < 0.01). For input ERα, MCF-7C + E2 vs. MCF-7C (^a^ *p* < 0.01), MCF-7S3 vs. MCF-7C (*** *p* < 0.001). For IP-ERα, a control that IP was successful (MCF-7C + E2 vs. MCF-7C ^a^ *p* < 0.001, MCF-7S3 + E2 vs. MCF-7S3 ^b^ *p* < 0.001, and MCF-7S3 vs. MCF-7C (*** *p* < 0.001). For IP-Sirt3, a confirmation that there is no interaction between ERα and Sirt3. For IP-p53, two-way ANOVA revealed a significant interaction effect between Sirt3 and treatment on the p53 level F (1,4) = 64.47, *p* = 0.001, partial *η*^2^ = 0.942; lower in the control (*** *p* < 0.001) and the E2-treated (** *p* < 0.01) MCF-7S3 vs. MCF-7C. In MCF-7S3, lower in the control vs. the E2-treated cells (^c^ *p* = 0.003). Representative blots are shown (*n* ≥ 3).

Due to the observed higher expression of p53 in MCF-7S3 cells, we hypothesized that Sirt3 can contribute to the lower proliferative capacity of the cells by increasing the p53 level. It is known that ERα binds p53 and represses its function [35]. Since we observed no interaction between Sirt3 and p53 (Supplementary Figure S1), we tested to determine if Sirt3 affects p53 indirectly by altering the crosstalk between ERα and p53. Using coimmunoprecipitation, we demonstrated that in the MCF-7S3 cells ERα binding to p53 was markedly reduced as compared with the MCF-7C cells (*p* < 0.001) and E2 addition partially reverted this interaction (Figure 7). These results indicate that Sirt3-induced disruption of the ERα–p53 interaction is partially reverted by E2 addition.

Collectively, on the one hand, the results show that Sirt3 improved the antioxidative response and mitochondrial fitness of the MCF-7 cells. On the other hand, it increased cytosolic ROS, DNA damage, along with p53, AIF, and ERα expression. Moreover, Sirt3 disrupted the p53–ERα interaction resulting in attenuation of tumor-promoting properties and proliferative effect of E2 in MCF-7 cells.

## 4. Discussion

The significance of Sirt3 in breast cancer cells lies in the fact that only 23% of normal breast tissue shows to be negative for Sirt3 expression, whereas even up to 72% of in situ breast lesions and 74% of invasive lesions are negative for the expression of Sirt3 [14]. Previously, we showed the tumor-suppressive role of overexpressed Sirt3 in human MCF-7 breast cancer cells, which are characterized by low Sirt3 expression [8]. In this study, we investigated if the tumor-suppressive effect of Sirt3 is mediated through the ERα signaling pathway in MCF-7 cells [3]. In this study, we report that a mitochondrial protein Sirt3, despite improving the mitochondrial function, induces DNA damage and tumor-suppressive factors, affects p53 by disruption of the ERα–p53 interaction, reduces response of the MCF-7 cells to E2 treatment, and consequently inhibits the migration and clonogenic capacity of the cells. 

The expression of ERα can be regulated by different cellular factors and mechanisms (reviewed by [36]) and, in our study, we found that there is significant upregulation of ERα expression in Sirt3-overexpressing cells (Figure 1B,C). Furthermore, Sirt3 silencing caused downregulation of ERα expression (Figure 1D), indicating the involvement of Sirt3 in the ERα expression level. Since Sirt3 is a mitochondrial protein and ERα is mostly localized in the nucleus [37,38], it is unlikely that they interact. Some earlier studies proposed the regulation of a small fraction of ERα by estrogen in mitochondria (reviewed by [39]), but these claims are still controversial. We were also not able to confirm interaction between Sirt3 and ERα (Supplementary Figure S1). Moreover, confocal microscopy confirmed, in our model, that ERα, indeed, does not reside inside mitochondria, and Sirt3 does not reside in the nucleus (Figure 2) or cytoplasm, as was already shown in our previous study [8]. Thus, we hypothesized that overexpressed Sirt3 indirectly regulates the expression of ERα in MCF-7 breast cancer cells. It is known that ERα contains a nuclear localization signal for the transport into the nucleus, which happens 10 to 30 min after E2 stimulation (reviewed by [40]). Therefore, we investigated the nuclear level of ERα as an indicator of its E2-stimulated activation. The fractionation experiments indicated that both MCF-7C and MCF-7S3 cells have functional ERα because it was successfully shuttled into the nucleus upon E2 treatment (Figure 1E). ICI, a compound that impairs the dimerization of ER-α, an event that takes place after E2 binding and is essential for the nuclear localization of the receptor [41], led to receptor degradation (Figure 1C,E) and partially depleted its nuclear localization as judged by more cytoplasmic ERα (Figure 2). Altogether, these data confirmed that ERα in our system is activated by E2 and that ICI is a good control for analysis of the ER-dependent pathways.

Several studies have shown that E2 has the ability to increase mitochondrial function and biogenesis (reviewed in [42]), therefore, we explored whethe Sirt3 plays a role in E2-induced metabolic fitness of MCF-7 cells. As observed in Figure 3A,C, Sirt3 potentiated this inducing effect of E2 leading to increased metabolic activity, primarily as a result of the induced rise in SDH-A expression (Figure 3B) [26]. This is not surprising, considering that ERα is an essential estrogen receptor for most E2-mediated increases in respiratory chain proteins and antioxidant enzymes [43,44]. Furthermore, Sirt3 also induced expression of complex I (NDUFA9) and III (UQCRC2) and elevated mitochondrial potential (Figure 3C). The latter was further enhanced upon E2 addition, indicating that the E2-induced rise in mitochondrial potential is Sirt3 dependent.

Elevated ROS is a common hallmark of cancer progression and can activate oncogenic signals involved in cell proliferation [45,46], hence, we wanted to check the ROS status of our cell lines. In agreement with others [47], our results showed that E2 promoted mtROS production in MCF-7C cells, while ICI abolished this effect (Figure 4E), suggesting the involvement of the ERα receptor in the E2-induced production of mtROS. This is not surprising, since E2 plays a critical role in the development of breast cancer by altering the mitochondrial function and causing a shift towards higher mtROS levels [48]. Sirt3 is a mitochondrial protein involved in the regulation of mitochondrial oxidative pathways [28] and we tested to determine if it regulates E2-induced proliferation by affecting ROS levels. In Sirt3-overexpressing cells, we observed lower mtROS levels (Figure 4E) and hypothesized that this could be the result of a more efficient mitochondrial antioxidant enzyme system induced by Sirt3. The oxidative stress response is manifested through upregulated antioxidative enzymes such as SOD and Cat that are under the control of Nrf2, which is activated upon oxidative stress or increased ROS production (reviewed by [49]). Our results showed a significant increase of Nrf2, Cat, and SOD in the MCF-7S3 cells (Figure 4A–C), indicating that the Sirt3-induced antioxidative response is associated with enhanced metabolic activity of the cells. Contrary to the MCF-7C cells, the E2-treated Sirt3-overexpressed cells showed lowered levels of both cytosolic and mtROS (Figure 4D,E), suggesting that the E2-generation of ROS is attenuated by Sirt3. Since numerous data postulate that an increase in ROS generated after exposure to E2 contributes to the development of human breast cancer [50,51], our findings propose that Sirt3 can function as a tumor suppressor by activating antioxidative enzymes and attenuating the E2-induced ROS levels from both cytosolic and mitochondrial compartments. 

Although the Sirt3-overexpressing cells demonstrated improved mitochondrial function and antioxidative enzyme system response, the opposite effect was observed on their proliferation and migration. Control of proliferation by estrogens is a very complex process based on E2 binding to ERα and subsequent regulation of target genes and concomitant activation of G1 to S phase progression [16]. In the case of non-invasive ERα-positive MCF-7 cells that otherwise display reduced expression of Sirt3 protein [52], the overexpression of Sirt3 diminished their growth, suggesting that Sirt3 inhibits their tumorigenic properties [8]. Furthermore, this inhibitory effect of Sirt3 is enhanced in the white DMEM regardless of E2 treatment (Figure 5A–D), indicating again that Sirt3 attenuates the proliferative effect of E2 on these cells. Interestingly, the observation that Sirt3-overexpressing cells have higher metabolic activity (Figure 3A) seems opposite to the fact that they show lower proliferation (Figure 5). However, cell cycle arrest does not necessarily result in metabolic dysfunction. On the contrary, some stressors can even increase the metabolic activity [53]. The Sirt3-mediated inhibitory effect on E2-induced proliferation was also supported by the analysis of DNA synthesis where we showed that E2 promotes DNA synthesis to a lesser extent in the MCF-7S3 cells as compared with the MCF-7C cells (Figure 5E). ICI decreases BrdU incorporation irrespective of Sirt3, confirming previous findings of the anti-proliferative effect of ICI by inhibition of DNA synthesis [53]. 

The mechanism of the Sirt3 effect on the reduction of clonogenic capacity is still not clear but involves DNA damage accumulation observed as an increase in γH2AX phosphorylation. The increased DNA damage was accompanied by a higher frequency of micronucleated cells in Sirt3-overexpressed cells, which are characterized by small, extra-nuclear chromatin bodies that arise in dividing cells due to chromosome aberrations or genome mutations (reviewed by [30]), and therefore are used as an indicator of genomic instability. In addition to the observed higher DNA damage and more micronucleated cells, the Sirt3-overexpressed cells also showed lower migration capacity. Some of these effects were partially rescued by E2 addition (Figure 6 and Supplementary Figure S2). These observations indicate that Sirt3 overexpression is associated with excessive genomic instability of MCF-7 cells, which can be alleviated by E2 treatment but only to a limited extent. However, this is not sufficient to rescue tumorigenic properties of these cells.

Since, in our earlier study, we observed higher levels of p53 in MCF-7S3 cells [8], we hypothesized that Sirt3 contributes to lower cellular proliferative capacity by increasing the p53 level. The upregulation of p53 and the tumor-suppressive effect of Sirt3 was shown also in other types of cancers [54,55]. The p53 protein is a transcriptional regulator and tumor suppressor that activates target genes for cell cycle arrest, apoptosis, and DNA repair, and is stabilized within the nucleus upon DNA damage or oncogenic signals (reviewed by [56]). Furthermore, p53 balances mitochondrial respiration by inhibiting the glycolysis and promoting OXPHOS. Our results showed that p53 is indeed upregulated in Sirt3-overexpressing cells (Figure 6A), but not through the interaction between Sirt3 and p53 (Supplementary Figure S1). Consistently, the apoptosis inducing factor (AIF), known to be transcriptionally upregulated by p53 [57], was also upregulated in Sirt3 clones. AIF is a mitochondrial flavoprotein harboring numerous functions for efficient oxidative phosphorylation and cytoprotective role in the mitochondria [58,59]. However, it has the opposite effect on cell survival upon translocation to the nucleus, where it serves as a potent pro-apoptotic trigger [60], resulting in large-scale DNA fragmentation [61]. This is also in line with the observed higher frequency of micronucleated cells in Sirt3-overexpressing cells (Figure 6C). Studies have shown that ERα directly binds to p53 and represses its function, thus, affecting p53-mediated cell cycle arrest [62]. Since ERα binding to p53 results in inactivation of p53, disruption of this interaction by Sirt3 indicates the possible mechanism of the tumor suppressive role of Sirt3 in breast cancer. Our results suggest that this disruption of the ERα–p53 interaction is partially rescued upon E2 addition (Figure 7). However, these E2-mediated effects are not sufficient for MCF-7S3 cells to reach the proliferation capacity of the control cells (Figure 5A–D). Furthermore, the observed overexpression of ERα (Figure 1C) correlates well with the increased level of p53 (Figure 6A), which is associated with inhibition of cellular growth [63]. Finally, while p53 can enhance metabolic activity by promoting OXPHOS [56], at the same time it inhibits cell cycle progression and causes a suppressive effect on growth of cancer cells.

## 5. Conclusions

In conclusion, based on the results from this study, we report that Sirt3, despite improving the mitochondrial function of MCF-7 breast cancer cells, reduces their response to E2, affects p53 by disruption of the ERα–p53 interaction, and inhibits clonogenic cell growth. Furthermore, the tumor suppressive effects of Sirt3 could be partially reversed by E2 treatment, but this is not sufficient to rescue the full tumorigenic potential of these cells. Therefore, because the majority of breast cancers are negative for the expression of Sirt3, we conclude that by reverting its expression in MCF-7 cells, breast cancer cell normalization could be induced. Thus, Sirt3 should be considered to be a potential therapeutic measure for treating E2-dependent breast cancers.

## Supplementary Materials

The following are available online at https://www.mdpi.com/2076-3921/9/4/294/s1, Table S1: Antibodies used in this study for Western blot analyses, Table S2: Antibodies used in this study for immunofluorescence analyses, Figure S1: Sirt3 does not interact with p53 or ERα in MCF-7 cells, Figure S2: E2 partially rescues reduced migration of MCF-7S3 cells.
