# Supplementary material for: Sirt3 Exerts Its Tumor-Suppressive Role by Increasing p53 and Attenuating Response to Estrogen in MCF-7 Cells
Source: Antioxidants (Basel). 2020 Apr 1;9(4):294. doi: 10.3390/antiox9040294 (PMC7222218; doi:10.3390/antiox9040294)
Supplement: Supplementary file 1 [file antioxidants-09-00294-s001.pdf]

Sirt3 Exerts Its Tumor-Suppressive Role by Increasing p53 and Attenuating Response to Estrogen in MCF-7 Cells

Marija Pinterić [1,†], Iva I. Podgorski [1,†], Marijana Popović Hadžija [1], Vedrana Filić [2] Mladen Paradžik [2,3], Bastien Lucien Jean Proust [1], Ana Dekanić [1], Ivan Ciganek [1], Denis Pleše [1], Dora Marčinko [1], Tihomir Balog [1] and Sandra Sobočanec [1,*]

1 Division of Molecular Medicine, Ruđer Bošković Institute, 10000 Zagreb, Croatia; mpinter@irb.hr (M. Pinteric); iskrinj@irb.hr (I.I.P.); mhadzija@irb.hr (M.P.H.); Bastien.Lucien.Jean.Proust@irb.hr (B.L.J.P.); adekanic@irb.hr (A.D.); iciganek@stud.biol.pmf.hr (I.C.); dplese@pharma.hr (D.P.); dora.marcinko@krka.biz (D.M.); balog@irb.hr (T.B.)

2 Division of Molecular Biology, Ruđer Bošković Institute, 10000 Zagreb, Croatia; Vedrana.Filic.Mileta@irb.hr (V.F.); Mladen.Paradzik@irb.hr (M. Paradzik)

3 Department Molecular Biotechnology and Health Sciences, Molecular Biotechnology Centre (MBC), University of Torino, 10124 Torino, Italy

* Correspondence: ssoboc@irb.hr; Tel.: +385-1-4561-172

† These authors contributed equally to this work

Received: 25 February 2020; Accepted: 30 March 2020; Published: date

Table S1. Antibodies used in this study for Western blot analyses.

Antibody	Dilution	Host	Manufacturer
Sirt3 (F-10, sc-365175)	1:500	Mouse	Santa Cruz Biotechnology, USA
ER-α (F-10, sc-8002)	1:500	Mouse	Santa Cruz Biotechnology, USA
GAPDH (ab9485)	1:5000	Mouse	Abcam, UK
H3 (ab1791)	1:3000	Rabbit	Abcam, UK
NDUFA9 (ab14713)	1:1000	Mouse	Abcam, UK
UQCRC (ab14745)	1:1000	Mouse	Abcam, UK
ATP5A (ab14748)	1:1000	Mouse	Abcam, UK
SDHA (ab14715)	1:1000	Mouse	Abcam, UK
AcSOD2 (ab137037)	1:1000	Rabbit	Abcam, UK
Catalase (ab1877)	1:1000	Rabbit	Abcam, UK
Nrf2 (ab31163)	1:1000	Rabbit	Abcam, UK
p53 (DO-1, sc-126)	1:2000	Mouse	Santa Cruz Biotechnology, USA
AIF (B-9, sc-55519)	1:500	Mouse	Santa Cruz Biotechnology, USA
gamma H2AX (phospho S139, ab11174)	1:8000	Rabbit	Abcam, UK
Anti-mouse (170-6516)	1:5000	Goat	Bio-rad, USA
Anti-rabbit (NA934)	1:5000	Goat	GE Healthcare, USA

Table S2. Antibodies used in this study for immunofluorescence analyses.

Antibody	Dilution	Host	Manufacturer
Sirt3 (F-10, sc-365175)	1:100	Mouse	Santa Cruz Biotechnology, USA
ER-α (ab16660)	1:500	Rabbit	Abcam, UK
gamma H2AX (phospho S139, ab11174)	1:5000	Rabbit	Abcam, UK
Alexa 488 (A-11001)	1:2000	Mouse	Thermo Fisher Scientific, USA
Alexa 594 (A-11012)	1:1000	Rabbit	Thermo Fisher Scientific, USA

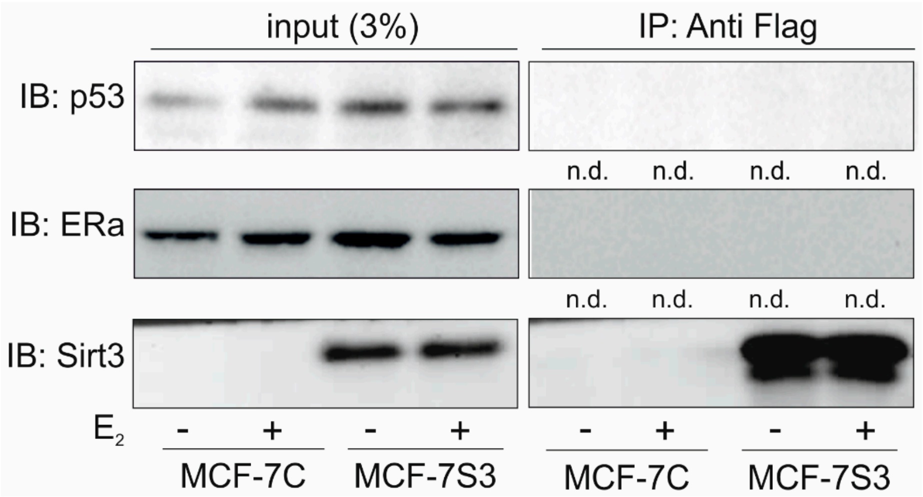

Figure S1. Sirt3 does not interact with p53 or ER-*α* in MCF-7 cells. Western blot analysis of coimmunoprecipitation experiment showing no interaction between Sirt3 and ER-*α* or p53.

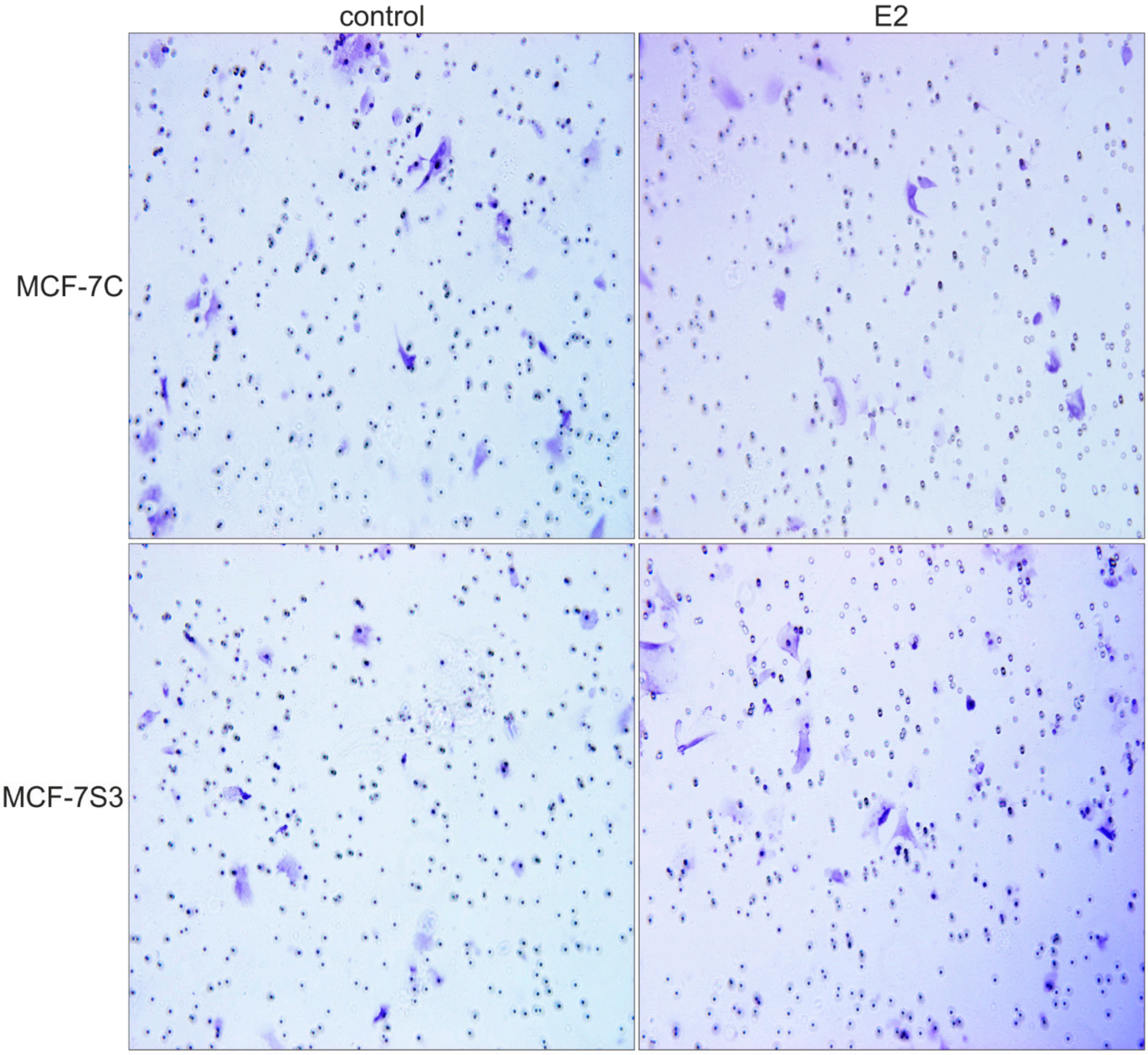

Figure S2. E2 partially rescues reduced migration of MCF-7S3 cells. Representative photographs of migrated MCF-7C and MCF-7S3 cells treated with E2. Samples are stained with crystal violet.
